# Vaccination timeliness among newborns and infants in Ethiopia

**DOI:** 10.1371/journal.pone.0212408

**Published:** 2019-02-19

**Authors:** Matthew L. Boulton, Bradley F. Carlson, Abram L. Wagner, Julia M. Porth, Berhanu Gebremeskel, Yemesrach Abeje

**Affiliations:** 1 Department of Epidemiology, School of Public Health, University of Michigan, Ann Arbor, Michigan, United States of America; 2 Department of Internal Medicine, Division of Infectious Diseases, University of Michigan Medical School, Ann Arbor, Michigan, United States of America; 3 Center for International Reproductive Health Training, University of Michigan, Ann Arbor, Michigan, United States of America; 4 Department of Public Health, St. Paul’s Hospital Millennium Medical College, Addis Ababa, Ethiopia; The Ohio State University, UNITED STATES

## Abstract

**Background:**

We characterize the risk factors for delayed polio dose 1, diphtheria-tetanus-pertussis (DTP) dose 1, pentavalent dose 1, and measles dose 1 in Ethiopian infants. We also examine the interaction between institutional delivery and demographic factors on the birth doses of the BCG and polio vaccines to better understand factors influencing vaccination.

**Methods:**

Using the 2011 Ethiopia Demographic and Health Survey, we calculated the distribution of the study population across different demographic and vaccination characteristics. We computed acceleration factors using a multivariable accelerated failure time model with a Weibull distribution to account for left and right censoring. For the birth doses, we further specified an interaction term between institutional delivery and every other *a priori* specified independent variable to test whether institutional delivery modifies sociodemographic disparities in vaccination timeliness.

**Results:**

Low wealth status, home delivery, and ethnicity are risk factors for delayed vaccination of polio 1, DPT 1, pentavalent 1, and measles 1. Religion is a risk factor for measles 1 vaccination delay and rural residence are risk factors for delayed DPT1 and polio 1 doses. For birth doses of polio and BCG, institutional delivery attenuated many sociodemographic disparities in vaccination delay, except for urbanicity, which showed rural dwellers with more delay than urban dwellers with an institutional vs home birth.

**Conclusions:**

Less delayed vaccination among children with institutional deliveries highlights the importance of perinatal care and the potential for promoting healthy behaviors to parents. Persistent disparities between urban and rural residents, even among those with institutional births, can be targeted for future interventions. Timely vaccination is key to prevention of unnecessary childhood mortality.

## Introduction

Immunization systems performance is often measured through vaccine coverage—the proportion of individuals who either have received a specific vaccine or have received all vaccine doses need to be considered fully immunized. Traditionally, full vaccination has comprised 1 dose Bacillus Calmette-Guérin (BCG) 3 doses polio vaccine, 3 doses diphtheria-tetanus-pertussis vaccine (DTP), and 1 dose measles vaccine. However, simply measuring immunization coverage at a point in time may ignore the timeliness of individual doses administered. Recent measles [1] and pertussis [2] outbreaks have occurred in the presence of relatively high overall levels of coverage and have instead been associated with children who were age eligible for a vaccination but who were delayed in receiving a dose. Curran et al. estimate that over 10% of pertussis cases in the U.S., and $1.3 million in costs, are tied to delayed pertussis vaccination [3].

According to the 2011 Demographic and Health Survey (DHS) in Ethiopia, only 24.3% of children between 12 and 23 months of age were fully immunized [4]. As vaccines are provided by healthcare workers at health facilities, healthcare access and utilization is important to consider when looking at predictors for complete immunization. Use of antenatal care [5] and postnatal care [4], delivery in health facilities [5–8], interactions with health workers [8], and shorter distance to health facilities [9] are all associated with higher levels of complete vaccination.

However, while vaccination coverage and defaulting are useful metrics, there can be substantial disparities between these measures and measures that consider timing of vaccine administration [10]. For example, analyses in the US of the Vaccine Safety Datalink, a consortium of 10 healthcare delivery organizations in 9 states, reveal that, among children born between 2005 and 2010, coverage for most vaccines was over 90% [11,12]. In contrast, less than half of children born between 2004 and 2008 had received all vaccine doses on time, as defined by recommendations from the Advisory Committee on Immunization Practices [13]. Information on vaccine timing from low- and middle-income countries is more limited, but is expected to be quite low, given the presence of low vaccine coverage. Research in The Gambia estimates that only slightly more than one third of children received all vaccinations on time [14]. In Uganda, this estimate of children who have been vaccinated on time ranges from 18% [10] to 45.6% [15].

Because timing of vaccination is an important measure of the ability to control individual and community susceptibility to vaccine-preventable diseases, and because predictors of vaccine timing may differ from other measures of vaccine coverage, we used data from the 2011 Ethiopia DHS to characterize the risk factors for delayed polio dose 1, DPT dose 1, diphtheria-pertussis-tetanus-*Haemophilus influenzae* type b-hepatitis B vaccine (pentavalent) dose 1, and measles dose 1 in Ethiopia. Finally, facility delivery has been linked to complete immunization in the literature [5–8,16]. To assess how facility delivery affects the influence of other predictors of vaccination, we model the interaction between institutional delivery and demographic factors on the birth doses of the BCG and polio vaccines.

## Methods

### Study population

Ethiopia is the 2^nd^ most populous African country after Nigeria, and had over 99 million residents in 2015 [17]. The country is ethnically, linguistically, and religiously heterogeneous: with more than 80 ethnic groups represented, the most populous are the Oromo (34.4%) and Amhara (26.9%), and although Orthodox Christians are a plurality of the population (43.5%), Muslims (33.9%) and Protestants (18.6%) are also sizeable [18–20]. As of 2014, Ethiopia is ranked 174 out of 188 countries for the Human Development Index [21].

There have been three DHSs conducted since 2000 in Ethiopia. The 2011 survey enrolled individuals from all 11 administrative regions of Ethiopia, using a two-stage cluster sample design. The first stage of the sampling scheme included census enumeration areas, which had been listed in the 2007 census. Within each census enumeration area, a map of all households was drawn, and a sample of households were chosen.

### Derived variables

The questions in this manuscript are derived from a women’s questionnaire that queried mothers between 15 and 49 years of age in the households sampled. Mothers were asked various questions about sociodemographic characteristics and their access to health care. They were also asked about the vaccination status of their children. For children with a vaccination card, the interviewer copied dates of any recorded vaccination onto the questionnaire; for children without a card, the interviewer asked the mother to recall if the child had received a vaccine. When a card with recorded dates was available, we calculated the age at vaccination by subtracting the date of vaccination from the date of their birth. Vaccination delay was the number of days between the date of vaccine administration and the recommended date (0 days (birth) for BCG and polio dose 0, 42 days for polio, DTP, and pentavalent dose 1, and 270 days for measles dose 1).

### Statistical analysis

We first calculated the distribution of the study population across different demographic and vaccination characteristics. The multivariable regression used an accelerated failure time model to account for substantial left censoring (i.e., children without vaccination cards who had a vaccine dose but unknown on which date) and right censoring (i.e., children who had not been vaccinated by the time of the study but who could have been after data collection was completed) [22,23]. Using the LIFEREG procedure in SAS version 9.4 (SAS Institute, Cary, NC, USA), we specified a model with a Weibull distribution. The Weibull distribution had the best fit, in its distribution of Cox-Snell residuals and in the -2 log likelihood measure, compared to a lognormal or gamma distribution. We present exponentiated β estimates, which are the acceleration factors (AFs) that correspond to the expected time to vaccination in the exposure versus reference group. All variables were *a priori* hypothesized to be related to vaccination timing, and are presented in Table 1. Additionally, we did not include antenatal care in the final models because of a high degree of missing data.

**Table 1:** Demographic description of 9264 children from 1 to 5 years of age in the 2012 Ethiopian DHS.

		Count	Weighted Proportion (95% CI)
Child’s sex	Male	4776	52.1% (50.7%, 53.6%)
	Female	4488	47.9% (46.4%, 49.3%)
Urbanicity	Rural	7521	85.0% (81.5%, 88.6%)
	Urban	1548	12.5% (9.0%, 16.1%)
	Not a de jure resident	195	2.4% (1.8%, 3.0%)
Wealth index	Poorest	2880	22.6% (19.7%, 25.5%)
	Poorer	1671	22.4% (20.2%, 24.7%)
	Middle	1464	20.4% (18.2%, 22.6%)
	Richer	1518	19.7% (17.3%, 22.1%)
	Richest	1731	14.9% (11.9%, 17.9%)
State	Tigray	966	6.5% (4.6%, 8.4%)
	Afar	929	1.1% (0.7%, 1.5%)
	Amhara	1046	22.8% (17.6%, 28.1%)
	Oromiya	1367	41.7% (34.7%, 48.7%)
	Somali	801	3.0% (1.8%, 4.2%)
	Benishangul Gumz	815	1.2% (0.8%, 1.6%)
	S.N.N.P.	1265	20.9% (16.1%, 25.8%)
	Gambela	683	0.4% (0.2%, 0.5%)
	Harari	523	0.2% (0.2%, 0.3%)
	Addis Ababa	314	1.8% (1.2%, 2.4%)
	Dire Dawa	555	0.3% (0.2%, 0.5%)
Ethnicity	Oromo	2251	37.2% (30.9%, 43.5%)
	Amhara	1600	25.6% (20.6%, 30.6%)
	Tigrie	958	6.4% (4.6%, 8.2%)
	Somalie	841	3.1% (1.9%, 4.3%)
	Affar	830	0.9% (0.6%, 1.3%)
	Other	2733	26.8% (21.8%, 31.8%)
Religion	Orthodox	2890	38.5% (32.9%, 44.1%)
	Protestant	1763	23.3% (18.4%, 28.2%)
	Muslim	4324	35.0% (28.8%, 41.1%)
	Other	283	3.2% (2.0%, 4.4%)
Number of people in household	1 to 3	1054	10.0% (8.8%, 11.2%)
	4	1348	14.9% (13.4%, 16.3%)
	5	1706	20.5% (18.8%, 22.3%)
	6	1599	17.5% (15.9%, 19.0%)
	7 and above	3557	37.1% (34.9%, 39.4%)
Number of children in household	1	3570	38.7% (36.0%, 41.3%)
	2	4137	46.1% (43.9%, 48.3%)
	3 and above	1557	15.3% (13.1%, 17.4%)
Mother working outside of home	No	4884	44.8% (41.7%, 47.9%)
	Yes	4290	55.2% (52.1%, 58.3%)
	Missing	90	
Child’s place of birth	Institutional	1316	10.1% (8.3%, 12.0%)
	Home	7928	89.9% (88.0%, 91.7%)
	Missing	20	
Number of antenatal care visits for newborn child	0	2925	57.4% (54.1%, 60.6%)
	1 to 3	1175	21.9% (19.8%, 24.0%)
	4 and above	1281	20.2% (18.0%, 22.5%)
	Don’t know	26	0.5% (0.1%, 0.9%)
	Missing	3857	

Notes: CI, confidence interval

For the birth doses (polio dose 0 and BCG), we further specified an interaction term between institutional delivery and every other independent variable to test whether institutional delivery modifies the degree to which the other sociodemographic variables impact vaccination timing.

We used an α level of 0.05 to assess significance and 95% confidence intervals (CI) to assess precision of the results. To estimate accurate standard errors and to make the results representative of the Ethiopian populace, the descriptive statistics used survey procedures, including clustering statements and weights. These survey procedures are not available for accelerated failure time models, and we simply used weights to produce unbiased estimates of the AFs; the CIs associated with these estimates may be unrealistically small because they do not account for the survey design.

### Ethical approval

This study was limited to previously collected, fully anonymized secondary data, and therefore is not under the purview of an institutional review board at the University of Michigan. Potential participants were read information about the study, including that it is a research study and participation is voluntary, and they provided verbal consent prior to any data collection.

## Results

A total of 9,264 children between 1 and 5 years of age were included in the dataset from all 11 administrative regions of Ethiopia (Table 1). The three largest ethnic groups represented were Oromo (37.2%), Amhara (25.6%), and Tigrie (6.4%), and the largest religious groups were Orthodox (38.5%), Muslim (35.0%), and Protestant (23.3%). The majority of children (85.0%) lived in rural areas and in relatively large households; most households with 2 (46.1%) or ≥3 (15.3%) children; overall, living with ≥7 people (37.1%) was the most common household size. Only 10.1% of mothers delivered in an institution, and over half (57.4%) had not received any antenatal care.

Vaccinations given in the first year of life were commonly delayed. In terms of birth doses, 71.3% of BCG and 51.1% of OPV 0 were given after 1 month. Infant vaccine doses were also typically delayed with 63.8% of DTP dose 1, 63.1% of Polio dose 1, and 68.5% of measles administered more than one month after the recommended date. Table 2 shows the proportion of individuals who received each vaccine, the proportion with a valid date on the card, and when the vaccine was administered relative to the recommended date. Vaccination coverage ranged from 15.3% for polio dose 0 to 76.7% for polio dose 1. Very few children had vaccination cards with a valid date(s) with a range of 16.5% for pentavalent dose 1 records to 30.3% for DTP dose 1 records. The median difference between the vaccination day and the recommended date ranged between two weeks (18.8 days for measles dose 1) to two months (64.1 days for BCG birth dose).

**Table 2:** Vaccination characteristics of 9264 children from 1 to 5 years of age in the 2012 Ethiopian DHS.

	Vaccine coverage		Children with a valid date on vaccination card^a^		Days of administration past recommended date^b^
	Count	Weighted % (95% CI)	Count	Weighted % (95% CI)	Median (95% CI)
BCG	5658	61.0% (58.0%, 64.0%)	1461	23.9% (21.5%, 26.3%)	64.1 (55.4, 72.9)
Polio dose 0	1884	15.3% (13.5%, 17.1%)	619	28.2% (24.5%, 31.9%)	35.2 (25.5, 45.0)
Polio dose 1	6807	76.7% (74.2%, 79.2%)	1657	20.6% (18.4%, 22.9%)	24.1 (15.8, 32.4)
DTP dose 1	5241	55.0% (51.8%, 58.3%)	1702	30.3% (27.4%, 33.1%)	27.3 (19.0, 35.6)
Penta dose 1	4218	45.6% (42.7%, 48.5%)	714	16.5% (14.2%, 18.8%)	23.3 (15.9, 30.6)
Measles dose 1	4878	52.1% (48.9%, 55.2%)	1251	21.7% (19.1%, 24.2%)	18.8 (13.6, 24.0)

Notes: BCG, Bacillus Calmette-Guérin; CI, confidence interval; DTP, diphtheria-tetanus-pertussis vaccine.

^a^ Among those with a dose of vaccine.

^b^ Among those with a valid date on vaccination card. The recommended date was 0 days (birth) for BCG and polio dose 0, 42 days for polio, DTP, and pentavalent dose 1, and 270 days for measles dose 1.

The fully adjusted associations between demographic characteristics and vaccination timeliness for four different vaccines are shown in Table 3. Urbanicity was related to timeliness of the administration of polio dose 1 and DTP dose 1; compared to those in rural areas, the expected time to vaccination was 0.74 times as high for polio dose 1 (95% CI: 0.60, 0.91) and 0.69 times as high for DTP dose 1 (95% CI: 0.54, 0.89). For most vaccines, there was a noticeable dose-response relationship between wealth and vaccination timeliness; for example, compared to individuals at the middle quintile of wealth, the estimated time to DTP dose 1 vaccination was 1.43 times higher among the poorest (95% CI: 1.19, 1.72), 1.15 times higher among the poorer (95% CI: 0.96, 1.37), 0.65 times as high among the richer (95% CI: 0.55, 0.78), and 0.52 times as high among the richest (95% CI: 0.41, 0.68). Ethnicity was also strongly related to vaccination timeliness; compared to the Amhara, time to vaccination was much longer among the Affar (AF ranging from 3.40 to 12.01), Somalie (AF ranging from 2.64 to 5.45), and Oromo (AF ranging from 1.63 to 2.12), whereas time to vaccination was less among the Tigrie (AF ranging from 0.22 to 0.53). After controlling for other socioeconomic variables, there were not strong associations between religion and vaccination timeliness, although for measles dose 1, Protestants (AF: 1.37, 95% CI: 1.21, 1.54) and Muslims (AF: 1.20, 95% CI: 1.08, 1.33) had a longer time to vaccination compared to Orthodox Christians. Having an institutional delivery was protective against vaccination delay for all vaccine doses considered; for example, compared to children delivered at home, children with an institutional delivery had only 0.59 times the length of time to DTP dose 1 vaccination (95% CI: 0.48, 0.73).

**Table 3:** The expected time to vaccination for four different vaccine series across different demographic groups, according to a fully adjusted accelerated failure time model with a Weibull distribution.

		Polio dose 1	DTP dose 1	Pentavalent dose 1	Measles dose 1
		AF (95% CI)	AF (95% CI)	AF (95% CI)	AF (95% CI)
**Child’s sex**	Male	ref	ref	ref	ref
	Female	0.91 (0.83, 1.00)	0.83 (0.74, 0.93)	0.84 (0.73, 0.97)	0.93 (0.86, 1.00)
**Urbanicity**	Rural	ref	ref	ref	ref
	Urban	0.74 (0.60, 0.91)	0.69 (0.54, 0.89)	0.91 (0.66, 1.25)	0.98 (0.84, 1.14)
	Not a de jure resident	1.46 (1.07, 1.98)	1.71 (1.14, 2.55)	1.09 (0.67, 1.79)	1.20 (0.94, 1.53)
**Wealth index**	Poorest	0.98 (0.85, 1.13)	1.43 (1.19, 1.72)	1.51 (1.19, 1.90)	1.36 (1.21, 1.53)
	Poorer	0.89 (0.78, 1.03)	1.15 (0.96, 1.37)	1.11 (0.89, 1.39)	1.08 (0.97, 1.21)
	Middle	ref	ref	ref	ref
	Richer	0.62 (0.54, 0.72)	0.65 (0.55, 0.78)	0.66 (0.53, 0.83)	0.85 (0.76, 0.95)
	Richest	0.57 (0.46, 0.70)	0.52 (0.41, 0.68)	0.65 (0.47, 0.90)	0.58 (0.50, 0.68)
**Ethnicity**	Oromo	1.69 (1.46, 1.95)	2.12 (1.77, 2.54)	1.76 (1.40, 2.23)	1.63 (1.46, 1.83)
	Amhara	ref	ref	ref	ref
	Tigrie	0.53 (0.43, 0.65)	0.22 (0.17, 0.27)	0.53 (0.39, 0.71)	0.53 (0.46, 0.60)
	Somalie	5.45 (3.89, 7.64)	5.41 (3.38, 8.66)	4.14 (2.30, 7.44)	2.64 (1.97, 3.54)
	Affar	8.05 (4.36, 14.86)	11.54 (4.35, 30.65)	12.01 (3.48, 41.48)	3.40 (1.92, 6.01)
	Other	1.47 (1.25, 1.73)	1.07 (0.88, 1.30)	0.68 (0.53, 0.87)	1.03 (0.91, 1.16)
**Religion**	Orthodox	ref	ref	ref	ref
	Protestant	1.21 (1.03, 1.41)	1.05 (0.86, 1.27)	1.02 (0.80, 1.31)	1.37 (1.21, 1.54)
	Muslim	1.02 (0.89, 1.17)	1.15 (0.97, 1.36)	1.18 (0.95, 1.46)	1.20 (1.08, 1.33)
	Other	1.13 (0.85, 1.50)	1.18 (0.82, 1.69)	0.89 (0.57, 1.38)	1.25 (1.00, 1.57)
**Number of people in household**	1 to 3	ref	ref	ref	ref
	4	0.83 (0.68, 1.01)	1.00 (0.79, 1.28)	0.99 (0.73, 1.36)	0.96 (0.82, 1.11)
	5	0.87 (0.72, 1.05)	1.10 (0.88, 1.39)	1.22 (0.91, 1.64)	1.10 (0.96, 1.27)
	6	0.71 (0.58, 0.86)	0.86 (0.68, 1.09)	0.95 (0.70, 1.28)	0.96 (0.83, 1.12)
	7 and above	0.78 (0.65, 0.94)	0.95 (0.76, 1.19)	0.97 (0.72, 1.29)	0.92 (0.80, 1.05)
**Number of children in household**	1	ref	ref	ref	ref
	2	0.82 (0.73, 0.91)	0.74 (0.65, 0.85)	0.71 (0.60, 0.85)	0.97 (0.89, 1.05)
	3 and above	1.25 (1.07, 1.45)	1.09 (0.90, 1.34)	1.05 (0.81, 1.34)	1.28 (1.13, 1.45)
**Mother working outside of home**	No	0.98 (0.89, 1.07)	1.17 (1.04, 1.32)	0.97 (0.84, 1.13)	1.11 (1.03, 1.20)
	Yes	ref	ref	ref	ref
**Child’s place of birth**	Institutional	0.80 (0.68, 0.96)	0.59 (0.48, 0.73)	0.83 (0.63, 1.08)	0.71 (0.63, 0.81)
	Home	ref	ref	ref	ref

Notes: CI, confidence interval; DTP, diphtheria-pertussis-tetanus vaccine; AF, acceleration factor.

Table 4 further examines the effect modification of institutional delivery on the other socioeconomic variables considered. For both birth doses considered—BCG and polio dose 0 –the interaction terms between institutional delivery and socioeconomic variables were significant for most, including urbanicity (for BCG), wealth index (for both birth doses), ethnicity (for both birth doses), religion (for BCG), number of people in household (for BCG), number of children in household (for polio dose 0), and mother working outside of home (for both birth doses).

**Table 4:** The effect modification of institutional delivery on the expected time to vaccination for BCG and polio dose 0, across different demographic groups, according to an accelerated failure time model with a Weibull distribution.

		Bacillus Calmette-Guérin (BCG)			Polio dose 0		
		Home delivery	Institutional delivery	Interaction p-value	Home delivery	Institutional delivery	Interaction p-value
		AF (95% CI)	AF (95% CI)		AF (95% CI)	AF (95% CI)	
Child’s sex	Male	ref	ref	0.1682	ref	ref	0.2893
	Female	0.83 (0.73, 0.94)	0.64 (0.45, 0.91)		0.81 (0.55, 1.22)	0.54 (0.29, 1.02)	
Urbanicity	Rural	ref	ref	0.0287	ref	ref	0.1853
	Urban	0.65 (0.48, 0.88)	0.32 (0.18, 0.57)		0.37 (0.16, 0.89)	0.14 (0.05, 0.39)	
	Not a de jure resident	1.47 (0.98, 2.21)	0.34 (0.09, 1.32)		3.51 (0.77, 16.03)	0.49 (0.06, 4.21)	
Wealth index	Poorest	1.38 (1.15, 1.66)	0.41 (0.14, 1.19)	0.0126	3.13 (1.63, 6.00)	0.29 (0.03, 3.19)	0.0314
	Poorer	0.99 (0.83, 1.18)	0.96 (0.36, 2.51)		2.05 (1.10, 3.83)	3.00 (0.24, 37.27)	
	Middle	ref	ref		ref	ref	
	Richer	0.76 (0.63, 0.91)	0.23 (0.10, 0.56)		0.36 (0.20, 0.64)	0.34 (0.04, 2.73)	
	Richest	0.81 (0.60, 1.09)	0.39 (0.16, 0.97)		0.48 (0.19, 1.18)	0.08 (0.01, 0.66)	
Ethnicity	Oromo	1.93 (1.58, 2.36)	0.80 (0.51, 1.25)	0.0062	7.60 (3.90, 14.80)	0.48 (0.21, 1.07)	<0.0001
	Amhara	ref	ref		ref	ref	
	Tigrie	0.23 (0.18, 0.30)	0.29 (0.15, 0.57)		0.71 (0.34, 1.46)	0.09 (0.03, 0.26)	
	Somalie	4.91 (3.06, 7.89)	1.44 (0.39, 5.34)		5.11 (0.99, 26.38)	0.95 (0.08, 11.31)	
	Affar	7.00 (2.99, 16.39)	3.13 (0.10, 102.68)		3.96 (0.22, 72.44)	1.01 (0.00, 348.28)	
	Other	1.11 (0.89, 1.38)	0.92 (0.55, 1.54)		2.02 (1.01, 4.04)	0.42 (0.17, 1.01)	
Religion	Orthodox	ref	ref	<0.0001	ref	ref	0.3623
	Protestant	1.35 (1.08, 1.67)	0.63 (0.38, 1.06)		1.34 (0.67, 2.68)	1.16 (0.46, 2.88)	
	Muslim	1.01 (0.84, 1.22)	1.56 (0.95, 2.56)		4.33 (2.27, 8.27)	1.78 (0.75, 4.23)	
	Other	1.06 (0.73, 1.56)	14.45 (3.27, 63.96)		5.19 (1.17, 23.00)	10.40 (0.80, 135.46)	
Number of people in household	1 to 3	ref	ref	0.0046	ref	ref	0.6576
	4	0.83 (0.63, 1.10)	0.44 (0.25, 0.77)		0.22 (0.08, 0.56)	0.26 (0.10, 0.68)	
	5	0.92 (0.71, 1.19)	1.09 (0.62, 1.91)		0.28 (0.11, 0.70)	0.37 (0.14, 0.98)	
	6	0.90 (0.69, 1.18)	0.38 (0.20, 0.73)		0.20 (0.08, 0.50)	0.53 (0.17, 1.63)	
	7 and above	0.76 (0.59, 0.98)	0.73 (0.39, 1.37)		0.30 (0.13, 0.74)	0.34 (0.11, 1.05)	
Number of children in household	1	ref	ref	0.3455	ref	ref	0.0007
	2	0.91 (0.79, 1.05)	1.27 (0.83, 1.95)		1.08 (0.68, 1.72)	5.37 (2.52, 11.44)	
	3 and above	1.25 (1.02, 1.54)	1.63 (0.69, 3.81)		0.95 (0.49, 1.84)	7.73 (1.50, 39.73)	
Mother working outside of home	No	1.26 (1.11, 1.43)	0.78 (0.55, 1.12)	0.0132	1.71 (1.12, 2.61)	0.69 (0.37, 1.29)	0.0193
	Yes	ref	ref		ref	ref	

Notes: BCG, Bacillus Calmette-Guérin; CI, confidence interval; AF, acceleration factor.

## Discussion

Ethiopia has made great gains in decreasing childhood mortality, cutting under-five mortality by two-thirds since 1990 and meeting its Millennium Development Goal target [24]. However, attaining the Sustainable Development Goal for reducing under-5 mortality from 59 deaths in 2015 [25] to 25 deaths per 1,000 live births in 2030 [26] will require improved child health services and ensuring that all children are receiving all doses of each vaccine included in the Expanded Program on Immunization at the appropriate time. This study found substantial delays in vaccine administration in Ethiopian newborns and infants when at their most vulnerable to severe illness and death from vaccine preventable diseases with wealth, urbanicity, ethnicity, and place of delivery all associated with vaccination timeliness.

We found that vaccine delays for this population ranged from about 2 weeks for the measles dose 1 vaccine to 2 months for the BCG vaccine. This is consistent with other African studies investigating delays in vaccination. A study from The Gambia found a median delay of 24 days for both BCG and the birth dose of polio [27]. In Uganda, investigators found a median delay of 24 weeks for the measles vaccine [10]. Further, median delays for pentavalent 1, pentavalent 3, and measles were 18 days, 21 days, and 26 days, respectively, in rural Kenya [28].

Wealth was found to be a significant predictor of vaccination timing in this study, with a longer expected time to vaccination among poorer families. This finding aligns with our current understanding of the relationship between wealth and immunizations from Uganda [15], Ghana [29], and 31 countries in sub-Saharan Africa [30]. Furthermore, though the relationship between timeliness and wealth has not been explored previously in Ethiopia, the literature does suggest that wealth is associated with other measures of vaccination, e.g., complete vaccination coverage and defaulting from the vaccination program [4,5,31–33]. The strength of the association between other socioeconomic variables and timeliness of vaccination for these birth doses differed depending on whether the child was born at home or at an institution. For example, for polio dose 0, we observe a dose-response relationship between wealth and vaccination timeliness for individuals born at home (e.g., compared to the middle quintile, the poorest had 3.13 times longer time to vaccination and the richest had only 0.48 times as high of delay). However, for children born at an institution, this disparity between the three lowest wealth quintiles is attenuated, although large bounds in the confidence interval could indicate small sample size. Similarly, for BCG administered to children born at home, the ethnic patterning of vaccination delay is similar to what we see for polio, DTP, penta, and measles dose 1: the Oromo, Somalie, and Affar have greater delay in vaccination. However, for children born at an institution, these ethnic disparities attenuate and there is no significant difference in the expected time to vaccination for children of these ethnic groups compared to the Amhara. This variation in vaccination timing by ethnic group could be the result of differences in health seeking behavior among different ethnic groups, which disappears when only comparing families utilizing institutional delivery. We do note that there is still a rural-urban discrepancy in the expected time to vaccination, with urban children taking less time compared to rural children, even for the group of children born at an institution. This association between wealth and timeliness may be due to challenges in healthcare access faced by poorer families and possible institutional barriers that individuals from certain ethnic groups face; while the vaccines are provided for free, families still must be able to afford transportation to the health center and lost productivity from taking their child to the clinic instead of working [5,31].

The relationship between urbanicity and vaccination timing appears to be less clear. Like this study, another study from Ghana [29], in addition to one using data from 31 countries in sub-Saharan Africa [30], found that vaccination statuses were worse in rural areas than urban areas. However, a studies from The Gambia [27] and Burkina Faso [34] found delayed receipt of most vaccines to be associated with living in urban areas. Each country represented by these different studies have varied political and social contexts. These varied results may be attributable to differences in governmental commitment to vaccine promotion programming. Countries in which the government has recently begun to focus on rural health promotion and vaccination efforts may show poorer vaccination outcomes as these programs are still being implemented while countries with well-established rural outreach immunization efforts may show better vaccination outcomes in their rural communities. The majority of studies from Ethiopia indicate living in urban areas is associated with positive vaccination outcomes, such as increased likelihood of complete vaccination and decreased likelihood of defaulting from vaccination programs [4,8,16,31,35]. In Ethiopia, urban areas historically have easier access to health services and better transportation available whereas rural health centers may face challenges with vaccine shortages, cold chain maintenance, attrition of the health care work force, and density of clinics relative to urban areas [4]. The introduction of the health extension program may have caused a recent increase in awareness of and access to vaccinations in rural areas, which may account for the mixed results in the literature [9].

This study found that, for the most part, institutional delivery attenuated the sociodemographic disparities in vaccination timeliness of BCG and polio dose 0. The impact on vaccination from ethnicity, religion, number of individuals living in the household, number of children living in the household, and maternal working status on timeliness were essentially null for women who delivered in a health facility, while many of these factors affected timeliness rates for women delivering at home. This suggests that as long as a woman can access a health facility for her delivery, her child is more likely to receive the BCG and polio birth doses, regardless of her social and economic characteristics. While there have been no studies to our knowledge that have examined this specific interaction between institutional delivery and other sociodemographic characteristics, this result is consistent with findings in other African nations that institutional delivery is an important predictor of vaccination timeliness [14,15,36]. Interestingly, this attenuating relationship does not exist for urbanicity; disparities between urban and rural women in timeliness are greater for those delivering in health institutions compared to those delivering at home. This disparity may be due to the challenges in vaccine availability, cold chain maintenance, and attrition of the health care workforce in rural health centers and should be a target for future interventions.

### Strengths and limitations

One limitation of the study is the cross-sectional nature of the Demographic and Health Survey, which complicates determination of causality. Although it is unlikely that demographic variables would be affected by a child’s vaccine timing, it could be that individuals of different demographic groups remember information about their child’s vaccination status differently, although the direction of this relationship is unclear. Families with missing data in one of the predictor variables were excluded from analysis and could bias results. Furthermore, the Demographic and Health Survey does not contain questions about parental knowledge, barriers to vaccination, or perceptions of vaccines. However, this analysis provides an important knowledge base for identifying and articulating the problem of vaccination timeliness, the mechanisms of which can be more thoroughly examined in future studies. This study also has important strengths. We used an analytical technique that incorporated left censoring, so we used information about when people were interviewed and what they recalled about their child’s vaccinations, to impute an understanding of when they could have possibly been vaccinated. This allowed us to include individuals with maternal recall information if no immunization card data was available, which increased our sample size and therefore the precision of our estimates and the power of our statistical tests. Additionally, using the Demographic and Health Survey data ensured that the study sample was representative of the entire country and the data were obtained from previously validated questionnaires.

## Conclusions

This research demonstrates that many Ethiopian children are receiving their vaccinations later than recommended, leaving them unnecessarily vulnerable to disease for extended periods. Moreover, the importance of urbanicity, wealth and other sociodemographic factors on vaccination timeliness—and the modification of these factors by place of delivery—demonstrates a need for increased promotion and utilization of institutional delivery. Future research should investigate parental knowledge, perceptions, and barriers to vaccination in Ethiopia to better understand why immunization is being delayed. It would also be valuable to investigate health worker knowledge of the recommended immunization schedule and vaccine timeliness and to examine the impact of knowledge on timeliness. It would be interesting to further explore this relationship between institutional delivery and timeliness by breaking down the institutional delivery and home delivery categories more specifically and examining how the specific health facilities and personnel involved in the birth affect vaccine timeliness, particularly in rural areas. Timely vaccination is an important tool for the decreasing preventable childhood mortality; the findings of this study can inform interventions designed to decrease delays in vaccination to ensure maximal protection from disease.
